# Application of NMR-based metabolomics and machine learning for non-invasive disease screening in dogs

**DOI:** 10.3389/fvets.2026.1830153

**Published:** 2026-06-30

**Authors:** Riccardo Finotello, Shao Thing Teoh, Hamed Nili, Mohammad Sepehri, Ylenia Cuzzupè, Simone Scoccianti, Fabio Procoli, Daniel C. Anthony, Livia Benigni, Mara Vittoria Alonzo, Islom B. Nazarov

**Affiliations:** 1 Department of Human Sciences, Link University, Rome, Italy; 2 Ospedale Veterinario I Portoni Rossi—AniCura Holding Italy S.r.l., Zola Predosa, Italy; 3 LatusPet Limited, Oxford, United Kingdom; 4 Department of Pharmacology, Medical Sciences Division, University of Oxford, Oxford, United Kingdom; 5 YouLiv4 Veterinary Imaging Referrals, London, United Kingdom

**Keywords:** blood biomarkers, cancer detection, cardiovascular disease, companion animals, machine learning, NMR metabolomics, veterinary diagnostics

## Abstract

**Background:**

Blood-based metabolomics is increasingly recognised as a powerful tool for disease detection in human medicine. However, its application in veterinary science remains limited.

**Objective:**

To evaluate the ability of an NMR-based metabolomics platform combined with machine learning to screen dogs for cancer, cardiovascular disease (CVD), and overall health status.

**Animals:**

Client-owned dogs were recruited from two sites. Of 156 animals enrolled, 139 remained after exclusions and were used for training and cross-validation of classification models.

**Methods:**

Blood samples were obtained from clinically healthy dogs and dogs with a range of diseases. Full blood count was performed, and serum metabolomic and lipoprotein profiling data were generated using NMR spectroscopy. Machine learning classifiers were trained to distinguish healthy from non-healthy dogs, and to further identify cancer and CVD cases. Model performance was evaluated by cross-validation and against null models with permuted class labels.

**Results:**

Models showed high discriminative performance for separating healthy from non-healthy animals (ROC AUC 0.916 ± 0.012; accuracy 86.5 ± 3.8%; sensitivity 81.7 ± 6.9%; specificity 87.5 ± 6.0%) and identifying pets with cancer (ROC AUC 0.911 ± 0.008; accuracy 83.5 ± 3.4%; sensitivity 86.5 ± 6.7%; specificity 82.4 ± 6.6%) or CVD (ROC AUC 0.924 ± 0.010; accuracy 90.0 ± 5.8%; sensitivity 85.6 ± 5.1%; specificity 90.6 ± 7.2%) from pets without the disease. Key predictive features included glutamine and creatine concentrations, lymphocyte count and percentage, platelet count and mean platelet volume (MPV), as well as lipoprotein cholesterol levels.

**Conclusion:**

This study provides the first evidence that NMR metabolomics combined with machine learning enables accurate, non-invasive, multi-disease screening in dogs, highlighting its potential for translation into routine veterinary practice for diagnosis and health monitoring.

## Highlights

- This study demonstrates, for the first time, that blood-based NMR metabolomics along with machine learning algorithms can accurately identify dogs with cancer or cardiovascular disease, as well as distinguish healthy animals from those with compromised health.
- Successful translation of metabolomics technologies into the veterinary field sets the foundation for broader adoption of precision diagnostics and preventative healthcare in companion animals.

## 1 Introduction

Early and accurate disease detection is central to clinical decision-making, timely intervention, and long-term outcome in veterinary medicine, yet diagnostic innovation in companion animals still lags behind human healthcare and often relies on invasive, costly, or poorly standardised approaches (1, 2). Dogs are particularly relevant in this setting because they develop many age-related disorders and are well established as comparative models of disease (3).

Among canine diseases, cancer and cardiovascular disease are major causes of morbidity and mortality (4). Cancer is a leading cause of death in dogs, but the canine tumour spectrum differs from that seen in people, with important differences in prevalence, biology, and breed association that support the need for species-specific diagnostic tools (5–10). At the same time, spontaneous canine tumours share several epidemiologic, biologic, and molecular features with human cancers, underpinning their value in comparative oncology (11–14). Cardiovascular disease in dogs is likewise biologically distinct from the human setting, being driven predominantly by myxomatous mitral valve disease (MMVD) rather than coronary atherosclerosis (15). Canine MMVD has both similarities to and differences from the human condition, again indicating that biomarkers developed in people cannot be assumed to translate directly to dogs (16–21).

Metabolomics offers a way to capture disease-associated biochemical changes in blood and has emerged as a promising approach for non-invasive diagnostics. Among available platforms, nuclear magnetic resonance (NMR) spectroscopy is particularly attractive because it is reproducible, quantitative, and able to profile a broad range of metabolites in a single assay (22–24). In human medicine, NMR-based metabolomics has been applied across several clinical areas, including cancer, cardiovascular disease, and inborn errors of metabolism (25, 26). In oncology, recent studies have demonstrated that NMR-derived metabolic signatures from blood and other biofluids can support early cancer detection, risk prediction, and tumour stratification across multiple cancer types, including breast and colorectal cancers (27–29). Similarly, in cardiovascular research, NMR-based metabolomics and lipoprotein profiling have been widely applied to characterize cardiometabolic risk, predict incident cardiovascular disease, and capture systemic metabolic perturbations associated with disease progression (30, 31). However, the diagnostic application of metabolomics in veterinary medicine remains comparatively limited, with most applications focusing on limited analytes rather than comprehensive profiling, and multi-disease screening platforms for companion animals remain largely undeveloped (32).

Machine learning provides an opportunity to integrate complex metabolic and haematological patterns into clinically useful classifiers. In this study, we applied the proprietary LatusPet platform, which combines serum NMR metabolomic profiling with machine learning, to address three aims: (1) distinguish healthy dogs from dogs with disease; (2) identify dogs with cancer; and (3) identify dogs with cardiovascular disease against a background of healthy and non-target disease states. Model performance was evaluated using cross-validation and null models with permuted labels. To our knowledge, this is the first study to assess high-throughput NMR-based metabolomics combined with machine learning for blood-based, multi-disease screening in dogs.

## 2 Methods

### 2.1 Ethics

This prospective study was carried out in Italy between 01 October 2024 and 03 October 2025. All procedures involving client-owned animals were conducted in accordance with relevant guidelines and regulations. The study was reviewed and approved by the Organismo Preposto al Benessere Animale (OPBA), University of Pisa, as non-experimental veterinary clinical practice under Italian Legislative Decree 26/2014. Approval was granted under delibera 50/2024 and delibera 51/2024 (issued 27/08/2024). Written informed consent was obtained from all pet owners prior to sample collection and participation in the study.

### 2.2 Study cohort

Serum samples were collected from client-owned dogs presenting for various medical conditions, as well as from healthy individuals. Based on clinical history and diagnostic findings, dogs were categorised into three groups: neoplastic, non-neoplastic, and healthy.

To be eligible for inclusion, a minimum dataset was required for all dogs, including signalment, physical examination, serum biochemistry, full blood count (FBC), electrolyte profile, urinalysis, and diagnostic imaging (including, at a minimum, thoracic radiography and abdominal ultrasonography).

Healthy dogs were recruited during routine wellness checks, vaccinations, elective neutering procedures, or as part of a blood donor programme. Exclusion criteria for this group included any evidence of active disease, abnormalities on clinical examination, or anomalies in laboratory or imaging findings. For blood donor dogs, a negative infectious disease screening panel was mandatory, in line with standard donor screening protocols and regional epidemiological risk. Screening included a rapid enzyme-linked immunosorbent assay (ELISA)-based test (SNAP^®^ 4Dx^®^ Plus Test, IDEXX Laboratories Italia S.r.l., Milan, Italy) for *Dirofilaria immitis* antigen and antibodies against *Borrelia burgdorferi* C6 peptide, *Ehrlichia canis/Ehrlichia ewingii*, and *Anaplasma phagocytophilum/Anaplasma platys*. In addition, all donor dogs were tested for Leishmania infantum using a serological ELISA assay.

Dogs were assigned to the neoplastic group if they had a confirmed diagnosis of malignant neoplasia based on cytological and/or histopathological evaluation, reviewed by a board-certified veterinary clinical pathologist and/or board-certified veterinary anatomic pathologist, respectively. Only treatment-naïve dogs were included. Dogs with a known history of prior glucocorticoid administration were excluded, based on available clinical history and information provided by referring veterinarians and owners. The presence of comorbidities did not constitute an exclusion criterion. Additional staging investigations were carried out based on tumour type and financial feasibility, as determined by the attending clinician. All cases were reviewed and approved for inclusion by a board-certified veterinary oncologist.

The non-neoplastic group comprised dogs diagnosed with non-oncological diseases. Diagnostic classification followed current best-practice standards (e.g., endocrine testing, echocardiography, electrocardiography, bacterial cultures), and all cases were evaluated and confirmed by a board-certified veterinary internal medicine specialist prior to group allocation.

Dogs with incomplete data on sex, breed, or age, or those lacking a definitive diagnosis, were excluded from the study.

### 2.3 Blood collection and sample processing

Blood samples were collected by qualified veterinary professionals via venepuncture using standard aseptic technique. Whole blood was drawn into serum and allowed to rest at room temperature for 30 min to clot. Samples were then centrifuged at 1,500 × *g* for 10 min at 4 °C to separate serum. The supernatant was aliquoted into pre-labelled cryovials. All samples were stored at −20 °C for up to 7 days, then transferred to −80 °C for subsequent batch analysis.

All handling, processing, and storage procedures were standardised across sites and timepoints to minimise pre-analytical variability. No freeze–thaw cycles were permitted prior to final metabolomics and haematological profiling.

### 2.4 Data acquisition

Each blood sample was aliquoted into two samples. The first sample consisted of whole blood collected into ethylenediaminetetraacetic acid (EDTA) tubes for FBC analysis. All haematological analyses were performed using the same automated haematology analyser (Sysmex XN-1000 V, Sysmex Europe SE, Norderstedt, Germany). Automated counts were complemented by manual evaluation of a blood smear performed by a clinical pathologist, including assessment of cell morphology and verification of automated results when appropriate. This yielded information on conventional haematological parameters, including absolute counts of the main blood cell populations and white blood cell subtypes, platelet indices, and haemoglobin-related variables. A total of 17 features were obtained for each sample and incorporated into the FBC dataset.

The second sample was sent to a central analytical centre for NMR data acquisition. Serum samples were thawed in batches to minimize the duration at room temperature, ensuring that the time between thawing and data acquisition did not exceed 9 h. For each sample, 200 μL serum was mixed with 400 μL NMR buffer (final: 10% D₂O, 200 μM maleic acid, 3 mM TSP). If precipitate formed, samples were vortexed and centrifuged at 3,000 × g for 5 min. The supernatant was transferred to a 5 mm NMR tube. NMR experiments were performed according to Bruker IVD specifications using a Bruker Avance IVDr 600 MHz instrument equipped with a PATXI ^1^H−^13^C−^15^N and ^2^H decoupling probe. Samples were maintained at 310 K during acquisition. Data were acquired using NOESY and CPMG pulse sequences with water suppression. Each spectrum was recorded with 32 scans. Spectra were processed with a 0.3 Hz exponential line broadening before Fourier transformation using Bruker’s TopSpin software. Baseline and phase correction were applied, and chemical shifts were calibrated to TSP at 0.0 ppm and Maleic Acid (ppm 6.2) was used as an internal reference. From the acquired spectral data, 38 small-molecule metabolites including alcohols, amines, amino acids and carboxylic acids were quantified by targeted integration of peaks in the 1D NOESY spectrum at specific chemical shift regions using the Bruker IVDr Quantification in Plasma/Serum B.I. Quant-PS^™^ software (*IVDr-metabolites* dataset). Representative NMR spectra for each quantified metabolite are provided in Supplementary Table 1. Additionally, NOESY spectral regions between 0.8–1 ppm and 1.2–1.4 ppm representing methyl and methylene groups of lipoproteins (33, 34) were binned and integrated to obtain information on lipid content (e.g., triglycerides, phospholipids and cholesterol) of different lipoprotein density subfractions (*NOESY spectra* dataset).

### 2.5 Data pre-processing

Each input data set (*FBC*, *IVDr-metabolite*, *IVDr-lipoprotein*) was separately normalized at the sample level using Probabilistic Quotient Normalization (35). Samples entirely missing one or more of the above-mentioned data sets were excluded. At the feature level, features with 30% or more missing values were excluded. Median imputation was performed for the remaining features with missing values.

Demographic and host-related variables including age, sex, breed, and reproductive status were retained as part of the dataset; age was included as an explicit model feature, without further normalization, scaling or transformation, whereas the remaining variables were not explicitly modelled or adjusted for, given the limited sample size and heterogeneity of the cohort.

### 2.6 Machine learning model development

Supervised classification models were trained to distinguish healthy, cancer or CVD status using decision tree–based ensemble methods, with hyperparameters for each model optimized using an in-house tuning pipeline. Models were implemented within a repeated stratified cross-validation framework, wherein 10 repeats of 10-fold cross-validation was performed. In each repeat, samples were partitioned into 10 folds with preserved class proportions; models were iteratively trained on 9 folds (90% of the data) and evaluated on the held-out fold (10%). Predictions from all folds were concatenated to obtain out-of-sample predictions for each repeat. Model performance was assessed using receiver operating characteristic (ROC) and precision–recall (PR) curves, with area under the curve (AUC) calculated per repeat and summarized as mean ± standard deviation across repeats. To assess baseline performance, null models were generated by repeating the same procedure with permuted class labels.

Feature selection was performed by ranking features according to their importance within the training data of each cross-validation split, followed by iterative reduction of the feature set. Model performance (ROC AUC) was evaluated as a function of the number of retained features using the same repeated cross-validation framework. The optimal feature set was defined as the smallest subset achieving maximal or near-maximal ROC AUC.

For classification metrics, decision thresholds were determined within each cross-validation repeat based on the Youden’s J statistic computed from training predictions. These thresholds were applied to the corresponding held-out predictions to assign class labels. Confusion matrices and derived metrics (accuracy, sensitivity, specificity) were computed per repeat and summarized as mean values across repeats.

### 2.7 Null model generation

Null models were obtained by randomly permuting class labels in the training data and retraining under identical cross-validation conditions. ROC and PR curves were generated to establish baseline performance.

### 2.8 Feature importance and univariate analysis

Univariate differences were assessed using the Wilcoxon rank-sum test (36, 37). We controlled for multiple comparisons by controlling the false discovery rate (FDR) via the Benjamini–Hochberg method (38).

### 2.9 Assignment of lipoprotein identities to NMR spectra

To identify lipoprotein subfractions corresponding to the NOESY spectral bins with high importances in the classification models, a set of predicted lipoprotein profiles including subclass particle numbers and lipid compositions was obtained from the NOESY spectra using the Bruker IVDr Lipoprotein Subclass Analysis (B.I. LISA^™^ software). This software integrates the methyl (0.8–1 ppm) and methylene (1.2–1.4 ppm) regions of the NOESY spectrum to predict total levels of cholesterol, free cholesterol, phospholipids, triglycerides, and apolipoproteins A1/A2/B100, as well as the distributions of these analytes in different lipoprotein density fractions. The density fractions are: high-density lipoprotein (HDL, density 1.063–1.210 g.cm^−3^), further subdivided into four subfractions (HDL-1 to HDL-4, in increasing density and decreasing size, respectively); low-density lipoprotein (LDL, density 1.09–1.63 g.cm^−3^), further subdivided into six subfractions (LDL-1 to LDL-6); intermediate-density lipoprotein (IDL, density 1.006–1.019 g.cm^−3^); and very low-density lipoprotein (VLDL, 0.950–1.006 g.cm^−3^), further subdivided into 6 subfractions VLDL-1 to VLDL-6. A partial least squares (PLS) regression model was constructed using NOESY spectral bin intensities as predictors (x-variables) and B.I. LISA-derived lipoprotein parameters as responses (y-variables). For each NOESY spectral bin, PLS coefficients for all lipoprotein parameters were examined, and the parameter(s) with highest PLS coefficients were selected as putative matched identity for that NOESY bin. Where appropriate, identity assignments are revised to better reflect known characteristics of dog lipoprotein profiles and composition as reported in literature (39).

## 3 Results

### 3.1 Study overview

A total of 156 client-owned dogs met the inclusion criteria and were enrolled in the study. Following exclusion of dogs with incomplete measurement (e.g., missing full blood counts), data from 139 animals were used for predictive model development. This population was subdivided into three groups: dogs diagnosed with cancer (*n* = 34), dogs with non-neoplastic diseases (*n* = 81), and clinically healthy controls (*n* = 24).

The oncology group comprised 34 dogs, including the following breeds: 7 mixed-breed dogs, 5 Labrador Retrievers, 4 Cocker Spaniels, 2 German Shepherd Dogs, 2 Golden Retrievers, 3 Maremma Sheepdogs, 2 Pit Bulls, 2 French Bulldogs and one each of Beagle, Bergamasco Sheepdog, Bullmastiff, Italian Cane Corso, Jack Russell Terrier, Pomeranian, Italian Hunting Dog, and Yorkshire Terrier. There were 20 females (18 neutered and 2 entire) and 14 males (8 neutered and 6 entire). The median age was 120 months (range: 24–180 months). Ten dogs were diagnosed with malignant epithelial neoplasms; of these, 6 had localised disease and 4 presented with regional and/or distant metastases. Lymphoma was diagnosed in 12 dogs, while one dog was affected by myeloid leukaemia and one by multiple myeloma. Three dogs had mast cell tumours (one localised, two with regional or distant metastases). Three dogs had oral mucosal melanoma (two localised, one with regional lymph node metastasis). Two dogs were diagnosed with mesenchymal tumours (one localised, one with visceral metastases). The remaining two dogs were diagnosed with glioma and neuroendocrine neoplasia, respectively.

The non-neoplastic group included 81 dogs. Breeds represented were: 20 mixed-breed dogs, 7 Chihuahuas, 6 Labrador Retrievers, 4 French Bulldogs, 4 German Shepherd Dogs, 2 each of American Staffordshire Terriers, American Bullies, Border Collies, Brittany Spaniels, Cane Corso, Maltese, Miniature Schnauzers, Poodles, Shih Tzus, and Yorkshire Terriers, and 1 each of Airedale Terrier, Australian Shepherd, Beagle, Cavalier King Charles Spaniel, Cocker Spaniel, Dachshund, Dalmatian, Dogue de Bordeaux, Golden Retriever, Jack Russell Terrier, Newfoundland, Pekingese, Pinscher, Pit Bull, Pug, Rottweiler, Siberian Husky, Spanish Galgo, Spitz, West Highland White Terrier. The median age was 102 months (range: 8–198 months). There were 36 females (12 entire and 24 neutered) and 45 males (34 entire and 11 neutered).

Across both oncology and non-neoplastic disease groups and considering both primary diagnosis and comorbidities, 7 dogs were affected by endocrine disorders, 6 by immune-mediated conditions, 37 by gastrointestinal disease, 16 by cardiovascular disease, and 27 were presented for obesity management.

The healthy control group consisted of 24 dogs. Breeds were as follows: 8 mixed-breed dogs, 4 Golden Retrievers, 2 Australian Shepherds, and 1 each of American Staffordshire Terrier, Border Collie, Bullmastiff, Cavalier King Charles Spaniel, Jack Russell Terrier, Leonberger, Maltese, Pinscher, Poodle, and Rottweiler. The median age was 22 months (range: 10–108 months). There were 16 females (15 entire and 1 neutered) and 8 males (7 entire and 1 neutered). All healthy dogs were assessed during routine wellness examinations and confirmed clinically normal based on physical examination, routine laboratory testing, and diagnostic imaging. Five dogs were regular blood donors.

### 3.2 Machine learning model based on multi-analyte profiling accurately identifies healthy dogs

**Figure 1:**
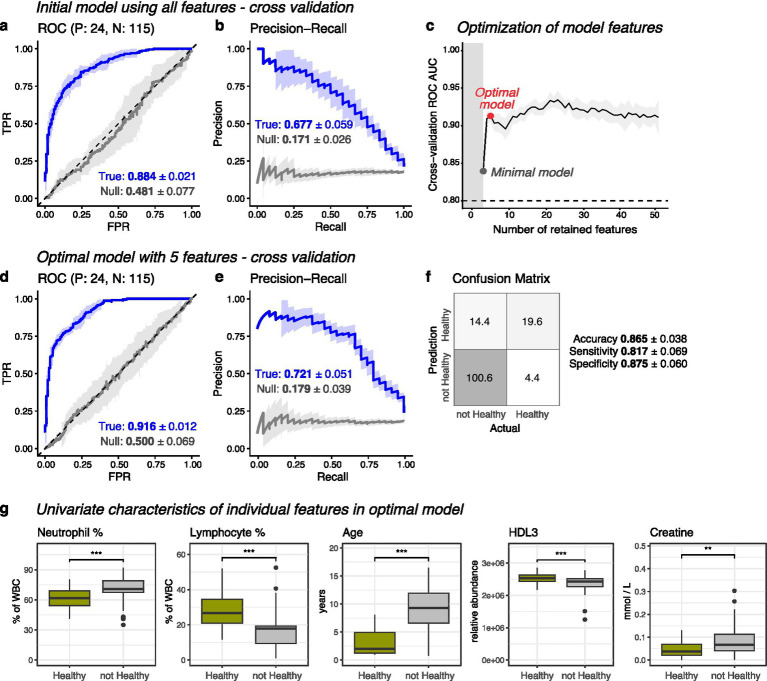
Machine learning model distinguishes healthy dogs from those with diverse disease states. **(a)** Receiver operating characteristic (ROC) and **(b)** precision–recall (PR) curves aggregated across repeated cross-validation (see Methods). Mean ROC and PR AUCs were 0.884 and 0.677, respectively, compared to substantially lower performance for null models. **(c)** Model performance (ROC AUC) as a function of the number of features. The optimal model was defined as the smallest feature set corresponding to a local maximum in ROC AUC (i.e., flanked by lower AUC at neighboring feature counts). Optimal performance was achieved with 5 features, while 3 features were sufficient to maintain AUC > 0.8. **(d)** ROC and **(e)** PR curves for the optimal 5-feature model, showing improved performance (mean ROC AUC = 0.916; PR AUC = 0.721) relative to the full model and null models. **(f)** Confusion matrix for the optimal model, with summary performance metrics (mean accuracy 86.5%, sensitivity 81.7%, specificity 87.5%; see Methods for calculation). **(g)** Univariate distributions of the five features included in the optimal model in healthy vs. non-healthy dogs. Boxplots show medians, interquartile ranges, and 2 × IQR whiskers; points indicate outliers. Statistical significance was assessed using Wilcoxon rank-sum tests with Benjamini–Hochberg correction. *, *p* < 0.05; **, *p* < 0.01; ***, *p* < 0.001; NS, not significant.

We first assessed the ability of the proprietary LatusPet machine learning model to distinguish healthy dogs from those diagnosed with cancer, cardiovascular disease, endocrine disorders, gastrointestinal disease, or obesity. After excluding animals with incomplete data, a binary classifier was trained on 139 dogs (24 healthy vs. 115 non-healthy, defined here as dogs with any diagnosed disease, including cancer, CVD or other diseases not modelled in the present study), corresponding to a class imbalance of ~1:4.8 (healthy: non-healthy). To account for this imbalance, model performance was evaluated using both ROC and precision–recall (PR) curves, the latter providing a more informative assessment under imbalanced class distributions. Using repeated cross-validation, the initial model achieved good discrimination (ROC AUC = 0.884 ± 0.021; PR AUC = 0.677 ± 0.059; Figures 1a,b), while null models with permuted class labels showed no discriminative power under the same CV analysis (ROC AUC = 0.481 ± 0.077; PR AUC = 0.171 ± 0.026).

To refine performance, we progressively removed low-importance features based on their ranking in the initial model. Optimizing for local maximum in ROC AUC while using as few features as possible, we found that an optimal model is obtained by retaining just 5 features (Figure 1c). Performance declined when the number of features was reduced below this threshold, although as few as three variables were sufficient to maintain an ROC AUC above 0.8. The optimized model showed improved ROC AUC (0.916 ± 0.012) (Figure 1d) as well as PR AUC (0.721 ± 0.051) (Figure 1e). Null models using the optimized feature set did not show any improvement (ROC AUC = 0.500 ± 0.069; PR AUC = 0.179 ± 0.039; Figures 1d,e). Across repeats, the true model achieved classification accuracy, sensitivity and specificity of 86.5 ± 3.8%, 81.7 ± 6.9% and 87.5 ± 6.0%, respectively (Figure 1f).

The 5 optimal features comprised two haematological parameters (neutrophil and lymphocyte percentages), an NMR-derived metabolite (creatine), a lipoprotein feature (HDL3 abundance), and age. Univariate comparisons confirmed significant group differences for all 5 variables (Figure 1g; Table 1), indicating consistency between model-derived importances and statistical testing.

**Table 1:** Top 5 important features for classification of healthy vs non-healthy dogs.

Feature	Unit	Non-healthy			Healthy			P_adjusted_
		q1	med.	q3	q1	med.	q3	
Neutrophil %	%	67.5	70.8	78.9	54.4	61.7	68.9	1.51E-4
Lymphocyte %	%	8.2	15.0	19.9	20.5	24.9	31.5	1.24E-5
Age	Years	6.66	9.55	11.98	1.21	1.84	5.05	4.55E-9
HDL3	Spectral intensity	2.3E+6	2.4E+6	2.5E+6	2.5E+6	2.5E+6	2.7E+6	2.00E-3
Creatine	mmol/L	0.0413	0.0690	0.1200	0.0206	0.0412	0.0761	7.68E-3

Summary statistics are shown for original, unprocessed values of dogs used for training of the Healthy classification model. Q1, med., q3 refer to 1st quartile, median and 3rd quartile, respectively. P_adjusted_ refers to the *p*-value adjusted for multiple comparisons using the Benjamini–Hochberg procedure.

### 3.3 Accurate classification of dogs with versus without cancer based on blood profiles and machine learning

**Figure 2:**
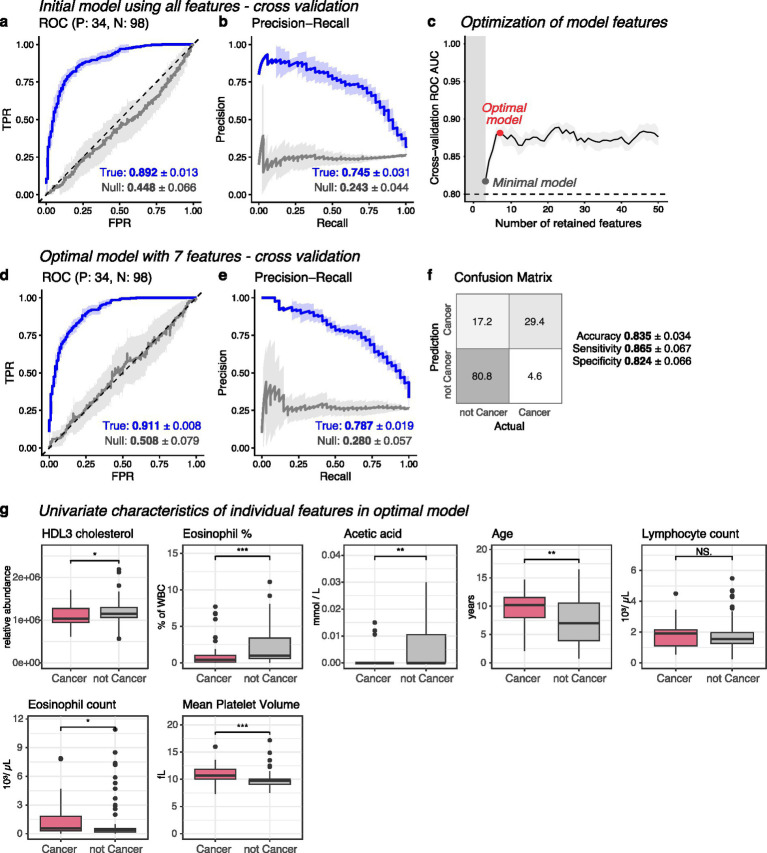
Machine learning model accurately identifies dogs with cancer among healthy and diseased populations. **(a)** Receiver operating characteristic (ROC) and **(b)** precision–recall (PR) curves aggregated across repeated cross-validation (see Methods). Mean ROC and PR AUCs were 0.892 and 0.745, respectively, compared to substantially lower performance for null models. **(c)** Model performance (ROC AUC) as a function of the number of features. The optimal model was defined as the smallest feature set corresponding to a local maximum in ROC AUC (i.e., flanked by lower AUC at neighboring feature counts). Optimal performance was achieved with 7 features, with 3 features sufficient to maintain AUC > 0.8. **(d)** ROC and **(e)** PR curves for the optimal 7-feature model, showing improved performance (mean ROC AUC = 0.911; PR AUC = 0.787) relative to the full model and null models. **(f)** Confusion matrix for the optimal model, with summary performance metrics (mean accuracy 83.5%, sensitivity 86.5%, specificity 82.4%; see Methods for calculation). **(g)** Univariate distributions of the seven features included in the optimal model in cancer vs. non-cancer dogs. Boxplots show medians, interquartile ranges, and 2 × IQR whiskers; points indicate outliers. Statistical significance was assessed using Wilcoxon rank-sum tests with Benjamini–Hochberg correction. *, *p* < 0.05; **, *p* < 0.01; ***, *p* < 0.001; NS, not significant.

We next developed a model to distinguish dogs with confirmed cancer diagnoses from all other dogs, including both healthy and with non-cancer diseases. The training set comprised 34 cancer cases and 98 non-cancer controls (including healthy dogs and those diagnosed with any non-neoplastic condition); 7 animals were excluded due to uncertain cancer status leaving 132 samples in total, corresponding to a moderate class imbalance of ~1:2.9 (cancer:non-cancer). In cross-validation, the initial model performed strongly (ROC AUC = 0.892 ± 0.013; PR AUC = 0.745 ± 0.031; Figures 2a,b) while null models failed to achieve meaningful discrimination (ROC AUC = 0.448 ± 0.066; PR AUC = 0.243 ± 0.044).

We next optimized the number of retained features as described in the previous section. We identified an optimal set of 7 variables yielding local maximum ROC AUC, while as few as three features were sufficient to maintain an ROC AUC above 0.8 (Figure 2c). The optimal model achieved ROC AUC = 0.911 ± 0.008 and PR AUC = 0.787 ± 0.019 (Figures 2d,e) in repeated CV, with classification accuracy 83.5 ± 3.4%, sensitivity 86.5 ± 6.7%, and specificity 82.4 ± 6.6% (Figure 2f). Null models with permuted class labels performed poorly (ROC AUC = 0.508 ± 0.079; PR AUC = 0.280 ± 0.057; Figures 2d,e), confirming that classification was highly significant and not due to random chance.

Unlike the healthy vs. non-healthy classifier, the optimal cancer model relied on FBC-derived variables (eosinophil %, lymphocyte count, eosinophil count, mean platelet volume [MPV]) while only HDL3 cholesterol, acetic acid and age made up the other optimal features. Univariate analysis revealed significant group differences for 6 of the 7 optimal features (Figure 2g; Table 2), including reduced HDL3 cholesterol and elevated MPV in cancer cases compared with controls, again highlighting good consistency between model-derived importance and statistical significance.

**Table 2:** Top 7 important features for classification of cancer vs non-cancer dogs.

Feature	Unit	Non-cancer			Cancer			P_adjusted_
		q1	med.	q3	q1	med.	q3	
HDL3 cholesterol	Spectral intensity	1.1E+6	1.3E+6	1.4E+6	1.0E+6	1.1E+6	1.3E+6	9.74E-3
Eosinophil %	%	0.465	1.600	4.200	0.100	0.315	0.838	1.88E-3
Acetic acid	mmol/L	0	0	0.0105	0	0	0	4.72E-2
Age	Years	4.4	7.1	10.8	8.1	10.3	11.6	6.67E-3
Lymphocyte count	10^3^/μL	1.11	1.38	2.11	1.11	1.92	2.16	3.10E-1
Eosinophil count	10^3^/μL	0.15	0.38	0.77	0.45	0.74	3.45	8.77E-3
MPV	fL	8.9	9.5	10.3	10.0	10.8	11.8	1.24E-3

Summary statistics are shown for original, unprocessed values of dogs used for training of the Cancer classification model. Q1, med., q3 refer to 1st quartile, median and 3rd quartile, respectively. P_adjusted_ refers to the *p*-value adjusted for multiple comparisons using the Benjamini–Hochberg procedure.

### 3.4 Robust identification of cardiovascular disease via machine learning model

**Figure 3:**
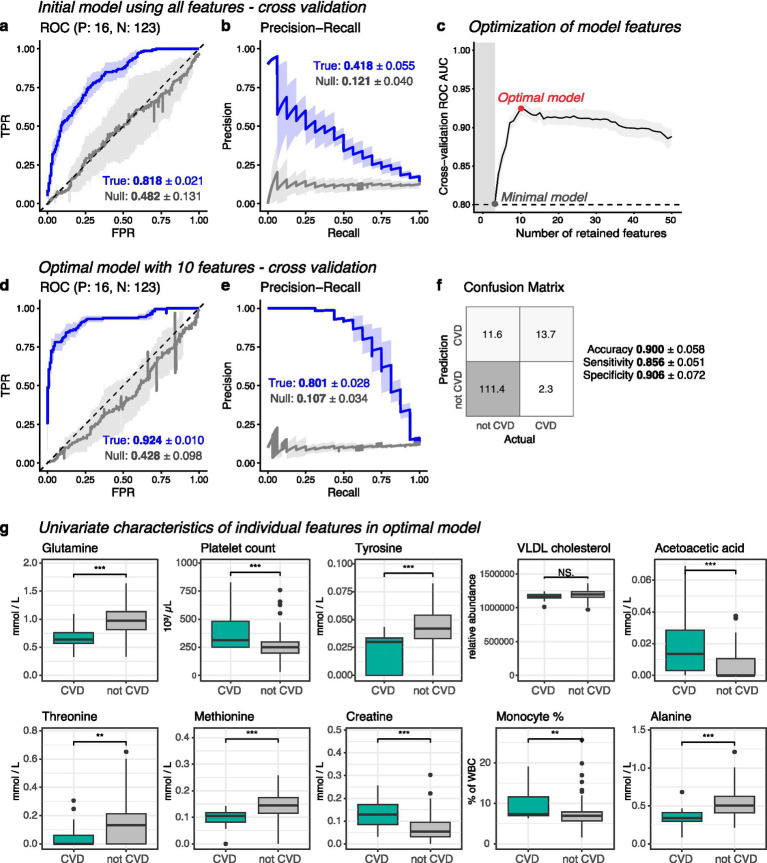
Machine learning model identifies cardiovascular disease (CVD) in dogs against a background of healthy and non-cardiac conditions. **(a)** Receiver operating characteristic (ROC) and **(b)** precision–recall (PR) curves aggregated across repeated cross-validation (see Methods). Mean ROC and PR AUCs were 0.818 and 0.418, respectively, compared to substantially lower performance for null models. **(c)** Model performance (ROC AUC) as a function of the number of features. The optimal model was defined as the smallest feature set corresponding to a local maximum in ROC AUC (i.e., flanked by lower AUC at neighboring feature counts). Optimal performance was achieved with 10 features, with 3 features sufficient to maintain AUC > 0.8. **(d)** ROC and **(e)** PR curves for the optimal 10-feature model, showing improved performance (mean ROC AUC = 0.924; PR AUC = 0.801) relative to the full model and null models. **(f)** Confusion matrix for the optimal model, with summary performance metrics (mean accuracy 90.0%, sensitivity 85.6%, specificity 90.6%; see Methods for calculation). **(g)** Univariate distributions of the ten features included in the optimal model in CVD vs. non-CVD dogs. Boxplots show medians, interquartile ranges, and 2 × IQR whiskers; points indicate outliers. Statistical significance was assessed using Wilcoxon rank-sum tests with Benjamini–Hochberg correction. *, *p* < 0.05; **, *p* < 0.01; ***, *p* < 0.001; NS, not significant.

We also assessed the performance of the model in identifying dogs with cardiovascular disease (CVD). The CVD model was trained on 16 CVD and 123 non-CVD samples (total 139 dogs), corresponding to a pronounced class imbalance of ~1:7.7 (CVD:non-CVD). As with the other models, cross-validation showed strong predictive power (ROC AUC = 0.818 ± 0.055; Figure 3a) although PR performance was noticeably lower (PR AUC = 0.418 ± 0.055; Figure 3b), consistent with the strong class imbalance and low prevalence of CVD, which reduces baseline precision despite good separability. Nevertheless, the true models greatly outperformed class permuted null models (ROC AUC = 0.482 ± 0.131; PR AUC = 0.121 ± 0.040; Figures 3a,b) indicating that the observed classification performance reflects genuine underlying biological signal rather than random chance.

From stepwise feature removal, the optimal model was found at 10 features and minimal model again required 3 features (Figure 3c). The optimal model displayed marked improvements over the initial model (ROC AUC = 0.924 ± 0.010; PR AUC = 0.801 ± 0.028), in contrast to the expected poor performance of randomly permuted null models using the same feature set (ROC AUC = 0.428 ± 0.098; PR AUC = 0.107 ± 0.034) (Figures 3d,e). Across repeats, classification accuracy, sensitivity and specificity of the true model were 90.0 ± 5.8%, 85.6 ± 5.1% and 90.6 ± 7.2% (Figure 3f).

The majority of features required for the optimal model were small molecules (glutamine, tyrosine, acetoacetic acid, threonine, methionine, creatine, alanine). Only 3 out of 10 features were lipoprotein or FBC parameters: platelet count, VLDL cholesterol, and monocyte %. Inspection of univariate feature distributions (Figure 3g; Table 3) revealed significant differences in all but one of the optimal model features between CVD and non-CVD dogs, reinforcing the relevance of these markers at both the multivariate modelling and univariate statistical levels.

**Table 3:** Top 10 important features for classification of CVD vs non-CVD dogs.

Feature	Unit	Non-CVD			CVD			P_adjusted_
		q1	med.	q3	q1	med.	q3	
Glutamine	mmol/L	0.813	0.974	1.112	0.571	0.638	0.759	3.84E-5
Platelet count	10^3^/μL	193	243	315	314	457	507	1.75E-4
Tyrosine	mmol/L	0.0323	0.0420	0.0540	0.0000	0.0300	0.0334	4.67E-4
VLDL cholesterol	spectral intensity	1.2E+6	1.4E+6	1.6E+6	1.4E+6	1.4E+6	1.6E+6	3.47E-1
Acetoacetic acid	mmol/L	0.0000	0.0000	0.0105	0.0090	0.0142	0.0664	6.13E-5
Threonine	mmol/L	0.0000	0.1305	0.2040	0.0000	0.0000	0.0604	1.50E-2
Methionine	mmol/L	0.116	0.146	0.173	0.083	0.105	0.117	6.13E-5
Creatine	mmol/L	0.035	0.056	0.100	0.087	0.131	0.180	4.67E-4
Monocyte %	%	5.7	6.9	8.3	7.3	9.3	13.4	1.19E-2
Alanine	mmol/L	0.403	0.503	0.623	0.297	0.340	0.413	1.75E-4

Summary statistics are shown for original, unprocessed values of dogs used for training of the CVD classification model. Q1, med., q3 refer to 1st quartile, median and 3rd quartile, respectively. P_adjusted_ refers to the *p*-value adjusted for multiple comparisons using the Benjamini–Hochberg procedure.

## 4 Discussion

### 4.1 Principal findings

Through this work, we sought to establish proof-of-principle for the application of advanced omics technologies to veterinary diagnostics, with the long-term goal of improving clinical outcomes and quality of life in companion animals. This study demonstrates, for the first time, the successful application of NMR-based metabolomic profiling combined with machine learning to screen dogs for cancer, CVD, and overall health status. Using the proprietary LatusPet platform, classification models accurately distinguished healthy animals from non-healthy animals spanning diverse disease states, and further differentiated dogs with cancer or CVD from healthy and non-target disease groups. These results establish a foundation for the development of non-invasive, blood-based diagnostic and health monitoring tools in veterinary medicine, where comparable innovations have historically lagged behind those in human healthcare.

Metabolomics has been extensively explored in human oncology and cardiology, with blood-based profiling showing promise for early detection, prognosis, and monitoring (40–44). However, its application to veterinary medicine, particularly systematic multi-disease screening in companion animals, remains largely unexplored. Our findings address this gap and support the broader translation of omics technologies into the veterinary field. Dogs develop a tumour spectrum that differs markedly from humans in prevalence, biology, tumour-type distribution, and breed association (5, 6, 10). These interspecies differences, together with the recognised comparative value of spontaneous canine tumours (11–14), underscore the importance of developing species-specific diagnostic platforms rather than directly extrapolating from human data.

Similarly, cardiovascular disease in dogs is predominantly characterised by MMVD, particularly in small breed, ageing animals (15). This is distinct from human cardiovascular disease, which is most commonly driven by atherosclerotic processes affecting coronary arteries. The ability of the proprietary LatusPet platform to identify canine CVD despite these aetiological differences suggests that metabolic signatures of systemic cardiac dysfunction can be captured effectively through blood-based analysis, independent of specific underlying mechanisms.

### 4.2 Comparisons to similar studies

Several commercial and experimental platforms have explored blood- or urine-based diagnostics for canine disease, most commonly cancer.

Volition Nu.Q^™^ quantifies circulating nucleosomes, which are elevated in cancer but can also rise in non-cancerous conditions such as inflammation, infection, trauma, and tissue injury, leading to potential false positives. In one study, the test achieved a sensitivity of 49.8% and specificity of 97% for cancer detection across all dogs, with an AUC of 68.7% (45). While highly specific, its modest sensitivity limits clinical utility, and the assay is restricted to cancer detection alone.

PetDx OncoK9^®^ Screen applies next-generation sequencing (NGS) of cell-free DNA combined with a proprietary machine learning algorithm. In pan-cancer detection, the test reached a sensitivity of 52.6% and specificity of 78.7% (46). Although the multivariate nature of NGS data has the potential to support diagnosis beyond cancer, this was not pursued, and overall performance is inferior to that achieved with the LatusPet platform.

Oncotect takes a novel approach, exploiting the chemotactic response of *C. elegans* to urinary volatile compounds. Cancer samples elicited increased chemotaxis, resulting in a reported accuracy of 87%, sensitivity of 85%, and specificity of 90% compared with healthy controls (47). While these results are impressive and comparable to those achieved by LatusPet, the reliance on a single readout restricts the assay to cancer detection and increases susceptibility to confounders such as hormonal cycles or systemic inflammation.

MI:RNA Cardiac Health Screening employs the expression of 15 serum or plasma microRNAs to detect MMVD. Using a penalized logistic regression model, the assay achieved sensitivity of 0.85, specificity of 0.82, and overall accuracy of 0.83 (48). Although this represents strong performance for cardiac disease detection, the LatusPet platform offers slightly superior accuracy and the additional advantage of detecting multiple conditions simultaneously.

A key limitation shared by most of these studies, except PetDx, is that models were trained to distinguish a specific disease from healthy dogs alone. Our internal findings suggest that this approach is insufficient for clinical deployment. Models trained as “disease vs. healthy” are prone to overfitting on the healthy class, leading to two major drawbacks: (i) the apparent disease classification may reflect separation from healthy controls rather than true disease-specific signatures, inflating performance metrics; and (ii) such models are not validated for distinguishing a target disease against a realistic background of other morbidities, which is the context of clinical decision-making.

By contrast, the LatusPet platform was trained using “condition vs. non-condition” models, ensuring robustness against diverse disease backgrounds. This design, combined with the integration of multi-modal, high-dimensional data, enables accurate simultaneous detection of cancer, cardiovascular disease, and overall health status. Building on this foundation, models for additional disease phenotypes are currently under development, further highlighting the multi-disease applicability of our high-dimensional dataset. To our knowledge, this represents the first demonstration of a veterinary diagnostic platform with such breadth, sensitivity, and specificity.

### 4.3 Interpretation of the key features

The overlap between features identified through univariate statistical testing and those selected by the machine learning models supports the robustness and biological relevance of the identified predictors. While more advanced interpretability methods such as SHAP values could provide additional insight, the current approach already captures key variables driving classification performance.

Mean platelet volume (MPV), one of the top features for cancer classification, is a marker of platelet activation and turnover, which are known to be altered in inflammatory, neoplastic, and cardiovascular states. Elevated MPV may reflect the pro-thrombotic and inflammatory environment commonly associated with cancer and CVD progression. In human disease, MPV has emerged as a potential biomarker for various cancers, including gastric, rectal, and head and neck cancers (49, 50). Studies have shown that MPV levels are often elevated in cancer patients compared to healthy controls, with some cancers showing decreased levels (49). MPV can be used for early diagnosis, monitoring disease progression, and assessing treatment response (51, 52). In dogs, MPV has been found to be increased in hematologic neoplasia and immune-mediated thrombocytopenia (53, 54). However, MPV can be influenced by various factors, including inflammation, medications, and other medical conditions. Thus, while MPV shows promise as a cancer biomarker here, it needs be evaluated alongside other the other markers for accurate assessment (55). The results presented here highlight that more research is needed to fully elucidate the role of MPV in cancer progression and metastasis (49).

Similarly, eosinophil counts, both absolute and percentage, emerged as important predictors. Eosinophils are implicated in tumour immunity and remodelling processes in various tissues, and shifts in their circulating levels may reflect systemic inflammatory responses to cancer or cardiovascular pathology. In humans, recent studies suggest that eosinophils may serve as a biomarker for cancer detection and prognosis. A large-scale analysis of the UK Biobank found an inverse association between eosinophil counts and overall cancer risk, with higher counts potentially offering protection against various cancer types (56). Several studies have reported associations between eosinophil levels and cancer outcomes, particularly in melanoma, lung cancer, and breast cancer (57–59). In colorectal cancer, tumour-associated tissue eosinophilia has been linked to favourable prognosis (60). Eosinophils may play both anti-tumorigenic and pro-tumorigenic roles, depending on the cancer type and microenvironment (61). While eosinophils show promise as a biomarker, their exact mechanisms in cancer progression and treatment response remain unclear, warranting further research (62, 63). Importantly, haematological features such as MPV and eosinophil counts are unlikely to be cancer-specific in isolation, as similar alterations can arise in a range of inflammatory, infectious, or non-neoplastic conditions. Rather, their value lies in capturing systemic host responses that, when integrated with metabolic and lipoprotein features in a multi-modal framework, contribute to improved classification performance without serving as independent diagnostic biomarkers.

The lipoprotein feature HDL3 cholesterol was also an important driver of the cancer prediction. Altered lipid profiles, including changes in LDL, VLDL, and HDL cholesterol levels, are associated with carcinogenesis and metastasis (64, 65). Cancer cells exhibit metabolic reprogramming, exploiting lipids for energy, membrane structure, and signalling (66, 67). The SREBP-1 transcription factor emerges as a key regulator of lipid metabolism in cancer, linking oncogenic signalling to metabolic alterations (68). Lipidomics techniques have advanced our understanding of cancer-specific lipid profiles, revealing potential biomarkers and therapeutic targets (69). Dysregulated lipid metabolism is now recognized as a hallmark of cancer, with ongoing research exploring lipid-lowering drugs and anti-lipid peroxidation treatments as promising anti-cancer strategies (70, 71).

Taken together, the cancer-associated features point toward a coordinated axis involving immune remodelling, platelet activation, and lipid metabolism. The combination of altered eosinophil and lymphocyte parameters suggests systemic immune reprogramming, potentially reflecting both tumour-driven inflammation and immune evasion mechanisms. Concurrently, increased MPV indicates heightened platelet activation, which is increasingly recognized as contributing to tumour progression through immune modulation, angiogenesis, and metastatic dissemination. The reduction in HDL3 cholesterol further supports a shift in lipid metabolism, consistent with the metabolic demands of proliferating tumour cells and systemic inflammatory signalling. These findings suggest that the model captures a composite host response to malignancy rather than tumour-specific markers alone.

In contrast to the cancer model, the CVD classifier is dominated by small-molecule metabolites, indicating a shift toward systemic metabolic dysregulation as the primary signal. Decreased serum glutamine, a key amino acid involved in nitrogen transport and energy metabolism, may reflect heightened metabolic demand or impaired gluconeogenesis in cardiac dysfunction (72, 73). In parallel, reduced methionine levels may reflect increased oxidative stress and disrupted methylation pathways, which are associated with cardiovascular pathology – for example, dogs with congestive heart failure have been found to display increased oxidative stress markers and decreased antioxidant defences (74). Overall, alterations in proteinogenic amino acids glutamine, methionine, alanine, tyrosine and threonine, as well as creatine, are consistent with previous reports of altered energy metabolism, amino acid reprogramming, and reduced renal function in dogs with MMVD (75). Increased concentrations of acetoacetic acid, a ketone body, suggest a shift towards alternative energy substrates, possibly secondary to reduced cardiac output or systemic metabolic adaptation to chronic heart disease, as dogs with MMVD exhibit up to 40% ATP deficit, disrupted substrate utilization, and oxidative phosphorylation (73). The coordinated changes in amino acids (glutamine, methionine, alanine, tyrosine, threonine) and energy-related metabolites (creatine, acetoacetate) are consistent with a state of impaired energy homeostasis and increased metabolic stress. This pattern aligns with the concept of cardiac disease as a systemic metabolic disorder, in which reduced cardiac output and mitochondrial dysfunction lead to compensatory shifts in substrate utilization, including increased reliance on amino acid catabolism and ketone body production. The relative lack of strong immune or lipoprotein features compared to the cancer model further supports the notion that metabolic reprogramming is the dominant signal captured in CVD.

In the model distinguishing healthy dogs from non-healthy dogs, creatine level as well as neutrophil and lymphocyte percentage featured prominently. Healthy dogs exhibit lower blood creatine, which may reflect kidney health as creatine is a known blood biomarker of kidney malfunction (76). Neutrophil and lymphocyte percentage were positively correlated with Healthy status, suggesting that a high leukocyte population may be a good marker of immune health. Alternatively, the tendency to measure higher levels of leukocytes in blood from healthy dogs may reflect the increased robustness in immune cells that have not yet become exhausted from fighting disease. Finally, HDL3 abundance was increased in healthy dogs; interestingly, this is opposite from the trend observed for HDL3 cholesterol in cancer samples highlighted above, suggesting that the abundance and lipid profile of HDL3 may be a useful indicator of health. Thus, the features identified by the model are not only statistically significant but also biologically meaningful, supporting the relevance of the metabolomic signatures captured by NMR profiling in this context. Collectively, the features distinguishing healthy from non-healthy dogs suggest that the model is capturing a state of systemic homeostasis characterized by balanced immune cell composition, preserved renal/metabolic function, and intact lipid transport. The prominence of neutrophil and lymphocyte proportions points toward immune equilibrium as a defining feature of health, while alterations in creatine and HDL3 may reflect early deviations in metabolic and lipoprotein homeostasis that precede overt disease. This aligns with the concept that health is not simply the absence of pathology, but a stable, multi-system physiological state.

In summary, the features identified across the three models converge on a limited number of biological processes, suggesting that the classifiers are capturing coordinated systemic responses rather than isolated analyte changes. Broadly, these processes include (i) immune cell redistribution and inflammatory signalling, (ii) lipid transport and lipoprotein remodelling, and (iii) alterations in energy and amino acid metabolism. Notably, the relative contribution of these processes differs between disease contexts, with immune and lipid-associated features dominating cancer classification, and metabolic reprogramming features being most prominent in cardiovascular disease.

### 4.4 Strengths and limitations

A key strength of this study lies in the rigorous model development pipeline, which incorporated cross-validation and the use of permuted null models to benchmark performance, as well as feature elimination to remove noisy features that do not contribute to classification performance. To the best of our knowledge, our models are also the first to take advantage of multi-modal inputs: unlike previous reports utilizing much smaller numbers of inputs from single modalities (45, 47, 48), we combined data not only from NMR metabolomics and lipoprotein profiling but also full blood counts, all obtained from a relatively non-invasive blood draw procedure. The rich information contained in our multi-modal, high-dimensional data also allows diagnosis of multiple conditions from a single data acquisition, as exemplified by our ability to classify Healthy, Cancer and CVD conditions all using the same dataset.

Nonetheless, certain limitations must be acknowledged. The sample size, although sufficient for proof-of-concept, remains relatively modest, and future work should seek to validate these findings in larger, prospectively collected cohorts. While the models achieved strong discrimination, the interpretability of certain features remains constrained by the complexity of metabolic interrelationships and potential breed-specific effects that were not explicitly modelled. An additional limitation relates to the heterogeneity of environmental and host-associated factors across the study population. As dogs were housed in non-controlled home environments, variability in diet, activity levels, and other lifestyle factors may have influenced circulating metabolic profiles and introduced additional noise or confounding into the dataset. Similarly, reproductive status was not explicitly modelled and may contribute to variation in both haematological and metabolic parameters. Another important consideration is the difference in age distribution between groups, with healthy dogs being substantially younger than disease cohorts. Although age was included as a feature within the machine learning models, it remains possible that age-related physiological changes contributed to the observed separation between healthy and non-healthy animals, potentially inflating classification performance. Future studies should therefore aim to incorporate tighter matching or stratification for age, sex, breed and reproductive status, as well as environmental factors and controlled dietary conditions where feasible, to more precisely disentangle disease-specific signals from broader physiological variation.

In addition, as with most metabolomics datasets, technical batch effects may arise from sources such as variation in sample preparation, instrument performance over time, or acquisition across different runs. We did not apply explicit batch effect correction, as several measurements were generated using standardized absolute quantification workflows and others represent per-sample clinical measurements less susceptible to batch structure; moreover, the limited sample size constrained the robust application of correction methods without risking overfitting. Importantly, model performance remained consistently high across repeated cross-validation despite folds not being stratified by batch, suggesting that batch effects did not dominate the biological signal captured. Nevertheless, residual batch effects cannot be excluded, and future studies with larger, independently processed cohorts will be important to systematically assess and mitigate such effects.

### 4.5 Future directions

Future work will focus on expanding the breadth of disease phenotypes captured, improving early detection sensitivity, and incorporating breed- and age-stratified modelling approaches. Longitudinal studies will be essential to assess whether the LatusPet platform can not only diagnose existing disease but also predict disease development prior to clinical manifestation, but also to evaluate its utility in monitoring treatment response and long-term patient outcomes. Integration of NMR-based metabolomics with other omics modalities, such as genomics and proteomics, may further enhance diagnostic accuracy and biological insight.

Although results presented here concern diagnosis of dog samples, the principles and technical concepts are easily applied to other animal species, and work is already underway to establish similar diagnostic models in cats. The translation of this platform into clinical veterinary practice has the potential to transform routine health screening in companion animals, improving early intervention opportunities and ultimately enhancing lifespan and quality of life.

## 5 Conclusion

This study provides the first demonstration of NMR-based metabolomic profiling combined with machine learning for disease screening and health monitoring in dogs. The proprietary LatusPet platform accurately distinguished healthy animals from those with cancer or cardiovascular disease, highlighting its potential as a non-invasive diagnostic tool in veterinary medicine. These findings pave the way for broader application of advanced omics technologies in companion animal health, offering new opportunities for early disease detection and improved clinical outcomes.

## Data Availability

The datasets generated and analysed during the current study are not publicly available owing to the requirements of the ethics consent but are available from the corresponding author for non-commercial academic use upon reasonable request. The machine learning models employ decision tree–based algorithms with hyperparameters optimized using a proprietary tuning pipeline. Details of the tuning procedure and specific hyperparameter configurations are not publicly disclosed for commercial reasons but may be made available to academic collaborators upon reasonable request and execution of a non-disclosure agreement (NDA).
